# Carotid intraplaque neovascularization and cerebral microbleeds: correlation assessed by plane-wave ultrasensitive flow imaging

**DOI:** 10.3389/fneur.2026.1816499

**Published:** 2026-07-31

**Authors:** Rongfeng Wang, Tingting Liu, Fei Wang, Jimei Xu, Shugang Cao, Mingwu Xia

**Affiliations:** 1 Department of Neurology, Hefei Hospital Affiliated to Anhui Medical University (The Second People’s Hospital of Hefei), Hefei, China; 2 Department of Neurology, The Second People’s Hospital of Hefei Affiliated to Bengbu Medical University, Hefei, China; 3 Department of Ultrasound, Hefei Hospital Affiliated to Anhui Medical University (The Second People’s Hospital of Hefei), Hefei, China

**Keywords:** carotid plaque, cerebral microbleeds, intraplaque neovascularization, ultrasensitive microvascular flow imaging, ultrasonography

## Abstract

**Objective:**

Carotid intraplaque neovascularization (IPN) is a marker of plaque vulnerability, whereas cerebral microbleeds (CMBs) are established markers of cerebral small vessel disease. However, the relationship between carotid IPN and CMB burden has not been well characterized. This study aimed to investigate the association between carotid IPN and the presence and burden of CMBs.

**Methods:**

This study was conducted at the Department of Neurology, Hefei Hospital Affiliated to Anhui Medical University, Hefei, China. Consecutive patients with carotid plaques identified by carotid ultrasonography were enrolled. Participants were categorized into two groups: the non-cerebral microbleeds (non-CMBs) group and the cerebral microbleeds (CMBs) group, based on susceptibility-weighted imaging (SWI) findings on brain magnetic resonance imaging (MRI). Carotid intraplaque neovascularization (IPN) was assessed using Angio Plane-Wave Ultrasensitive Flow Imaging (Angio PLUS) and categorized as low (grades 0–1) or high (grade 2). Spearman correlation analysis was used to assess the association between IPN grade and CMB burden. Logistic regression analyses were performed to identify factors independently associated with overall, lobar, and deep/infratentorial CMBs.

**Results:**

A total of 493 patients were included, among whom 175 (35.5%) had CMBs. Spearman’s correlation analysis revealed a positive correlation between IPN grade and CMB severity (rs = 0.351, *p* < 0.001). Multivariate logistic regression demonstrated that high-grade IPN was an independent risk factor for the presence of CMBs (OR = 2.793; 95% CI: 1.841–4.239; *p* < 0.001). Subgroup analysis revealed that high-grade IPN was associated with deep/infratentorial CMBs but not lobar CMBs.

**Conclusion:**

Carotid IPN demonstrates a positive correlation with the severity of CMBs. Furthermore, high-grade IPN is independently associated with the presence of overall CMBs as well as deep/infratentorial CMBs, although it does not exhibit a similar association with lobar CMBs.

## Introduction

Cerebral microbleeds (CMBs) are a characteristic imaging manifestation of cerebral small vessel disease (CSVD) and are associated with an increased risk of stroke, particularly intracerebral hemorrhage, as well as cognitive impairment (1–3). The prevalence of CMBs is approximately 18.7% among individuals aged 45 years or older, and their presence is associated with an increased risk of stroke, particularly intracerebral hemorrhage, which is strongly linked to lobar CMBs (4). In addition, CMBs exert a detrimental effect on cognitive function, with prominent impairments in orientation, attention, calculation, and delayed recall (5, 6). CMBs are considered markers of diffuse vascular and neurodegenerative brain injury and may result from small vessel wall damage related to vascular risk factors and *β*-amyloid deposition (7, 8).

Previous studies have suggested an association between carotid atherosclerosis and CMBs, and plaque instability has been identified as a potential risk factor for CMB burden (9, 10). Intraplaque neovascularization (IPN), which results from vasa vasorum proliferation during early atherosclerotic progression, contributes to intraplaque hemorrhage and plaque instability (11, 12). As an early marker of plaque vulnerability, IPN may provide additional value for risk stratification in carotid atherosclerosis. Despite emerging evidence linking carotid atherosclerosis to CSVD, the relationship between carotid IPN and CMB burden remains unclear.

However, conventional imaging techniques mainly evaluate carotid plaque morphology and composition, including lipid-rich necrotic core and intraplaque hemorrhage; these methods may be limited in detecting early plaque vulnerability (13). Angio Plane-Wave Ultrasensitive Flow Imaging (Angio PLUS) enables non-contrast visualization of intraplaque microvascular flow with high sensitivity, safety, and reproducibility, making it suitable for clinical evaluation (14). Therefore, this study aimed to investigate its association with the severity of CMBs and to explore its potential value as a noninvasive imaging marker related to cerebral microvascular injury.

## Methods

### Study design and patients

**Figure 1:**
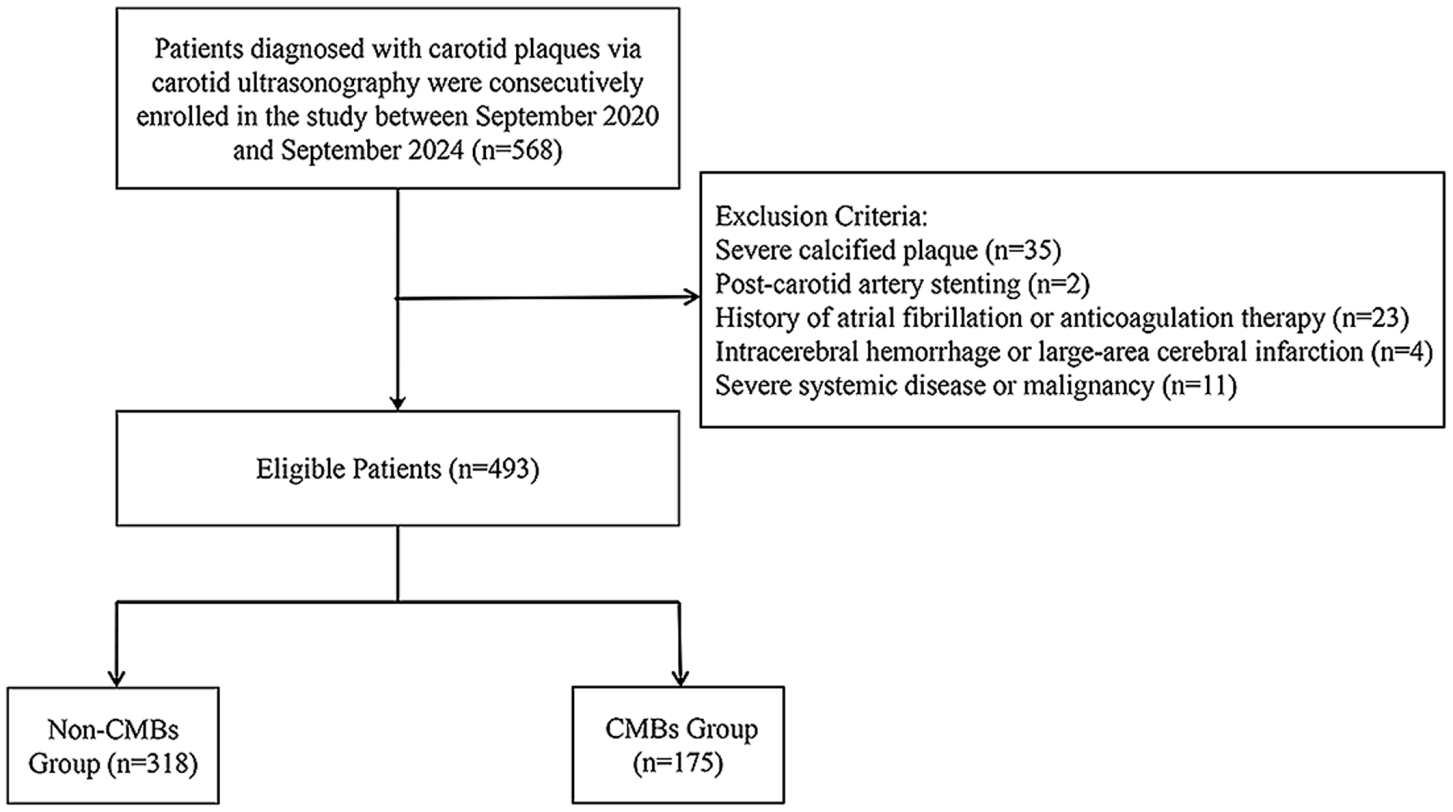
Flowchart of the study population.

This was a retrospective cross-sectional study. Patients were stratified into two groups according to the presence of cerebral microbleeds (CMBs): the CMB group and the non-CMB group (absence of CMBs). The non-CMB group was defined as patients without any cerebral microbleeds (0 CMBs). Patients admitted to the Department of Neurology at Hefei Hospital Affiliated with Anhui Medical University, and diagnosed with carotid plaques via carotid ultrasonography were consecutively enrolled in the study between September 2020 and September 2024. The inclusion criteria were as follows: (1) presence of carotid plaques detected by carotid ultrasonography; (2) completion of carotid IPN evaluation using the Angio PLUS ultrasound technique; and (3) completion of CMBs screening using brain MRI SWI sequence. Patients were excluded if they had: (1) severe carotid plaque calcification that significantly impaired IPN assessment; (2) a history of carotid artery stenting; (3) a history of atrial fibrillation or anticoagulant use; (4) comorbid intracerebral hemorrhage or large-area cerebral infarction; and (5) Patients with severe cardiopulmonary dysfunction, hepatic or renal dysfunction, or malignant tumours were excluded because these conditions may affect vascular remodeling, inflammatory status, and coagulation function, thereby potentially confounding the association between carotid IPN and CMBs. The detailed methodology of our participant selection process is depicted in Figure 1.

### Clinical data

Demographic data, including sex and age, were collected from all enrolled patients. Personal history variables included smoking history and alcohol consumption history. Vascular risk factors included hypertension, diabetes mellitus, and hyperlipidemia. Medical history and medication use included prior ischemic stroke, antiplatelet use, and statin use. Baseline blood pressure (systolic and diastolic) was recorded at hospital admission.

### Laboratory assessments

Fasting venous blood samples were collected on the morning after admission for complete blood count, coagulation profile, liver and renal function tests, and glycosylated hemoglobin (HbA1c). Levels of total cholesterol (TC), triglycerides (TG), high-density lipoprotein cholesterol (HDL-C), low-density lipoprotein cholesterol (LDL-C), fasting plasma glucose (FPG), creatinine, and fibrinogen were also recorded. The variables collected in this study were selected based on their established or potential associations with carotid atherosclerosis and CSVD. These variables were included to adjust for potential confounding factors in the relationship between carotid intraplaque neovascularization and CMBs.

### Diagnosis of carotid plaque and IPN assessment

Angio PLUS is an ultra-fast plane-wave-based ultrasound technique that enables non-contrast visualization of low-velocity microvascular flow and was performed using the AixPlorer ultrasound system (SuperSonic Imagine, France). Patients were placed in the supine position with the neck fully exposed, relaxed, and slightly rotated to the contralateral side. A standard carotid ultrasound examination was first conducted along the course of the carotid artery to measure intima–media thickness (IMT) and to document the location, size (maximum thickness and length), number, and echogenicity of plaques. Angio PLUS plane-wave ultrasensitive flow imaging was then applied to visualize and assess IPN within the target plaques. The number, location, morphology (e.g., punctate, short-linear), and flow-spectrum characteristics of intraplaque microvascular signals were recorded. IPN assessments were independently performed by two experienced sonographers who were blinded to all clinical information and brain MRI findings, including the presence and severity of cerebral microbleeds (CMBs). Any discrepancies were resolved by consensus.

IPN was graded according to the following criteria (15): Grade 0 indicated the absence of hyperechoic microvascular signals within the target plaque (no neovascularization); Grade 1 indicated several (<4) punctate or a few short-linear hyperechoic microvascular signals; and Grade 2 indicated diffuse (≥4) short-linear or strip-like hyperechoic microvascular signals within the plaque. Patients with IPN grades 0–1 were classified as the low-IPN group, whereas those with grade 2 were classified as the high-IPN group.

### Diagnosis and assessment of CMBs

**Figure 2:**
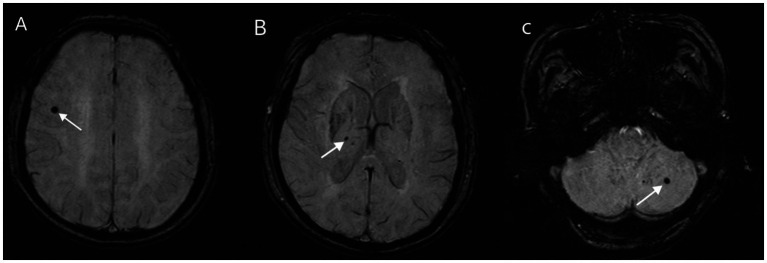
**(A)** Showed lobe CMBs; **(B)** Showed deep CMBs; **(C)** Showed infratentorial CMBs.

**Figure 3:**
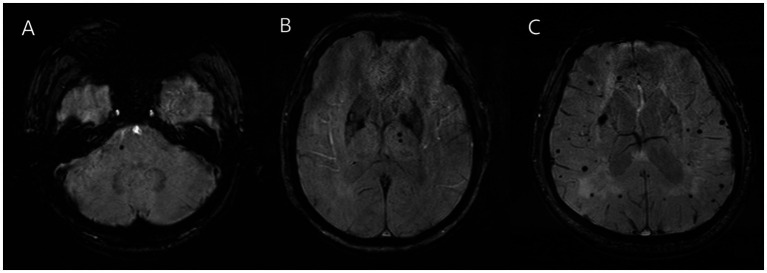
**(A)** Showed CMBs rated as grade 1; **(B)** Showed CMBs rated as grade 2; **(C)** Showed CMBs rated as grade 3.

MRI examinations were performed using a 1.5-T Siemens Avanto scanner (Siemens, Germany). Two neurologists independently and blindly evaluated all imaging data. CMBs were defined as round or ovoid hypointense lesions (2–5 mm, occasionally up to 10 mm) that appeared isointense on T1WI/T2WI/FLAIR and hypointense on SWI (3). The anatomical distribution of CMBs was classified according to the Microbleed Anatomical Rating Scale (MARS) developed by Gregoire et al. (16): Lobar CMBs: cortex, subcortical U-fibers, and subcortical white matter; Deep CMBs: basal ganglia, thalamus, internal capsule, external capsule, corpus callosum, and white matter within 10 mm of the lateral ventricles; Infratentorial CMBs: brainstem and cerebellum (Figure 2). For further analysis, CMB burden was categorized according to the total number of CMBs into four ordinal groups: 0 CMBs, 1 CMB, 2–4 CMBs, and ≥5 CMBs, as previously reported (17) (Figure 3).

### Statistical analysis

Statistical analyses were performed using SPSS version 24.0 (SPSS Inc., Chicago, IL, United States). The Kolmogorov–Smirnov test was used to assess data normality. Normally distributed continuous variables were expressed as mean ± standard deviation (x̄ ± s), whereas non-normally distributed variables were expressed as median with interquartile range (IQR, Q25–Q75). Comparisons between groups were conducted using the Student’s *t*-test or Mann–Whitney U test. Categorical variables were presented as frequency and percentage (%) and compared using the χ^2^ test or Fisher’s exact test. Spearman’s rank correlation analysis was used to examine the correlation between IPN grade and CMBs severity. Multivariate binary logistic regression analysis was performed to calculate odds ratios (ORs) and 95% confidence intervals (CIs) for identifying independent predictive factors. A two-tailed *p* value < 0.05 was considered statistically significant.

## Results

### Baseline characteristics

A total of 493 patients were included in the final analysis. Among them, 274 (55.6%) were male and 219 (44.4%) were female, with a mean age of 63.28 ± 5.83 years. There were 230 (46.7%) patients in the low-IPN group (grades 0–1) and 263 patients (53.3%) in the high-IPN group (grade 2). CMBs were identified in 175 patients (35.5%), including 37 (21.1%) with grade 1, 79 (45.2%) with grade 2, and 59 (33.7%) with grade 3 CMBs. Regarding anatomical distribution, CMBs were confined to deep regions in 28 patients (16.0%), infratentorial regions in 12 patients (6.9%), and lobar regions in 58 patients (33.1%), while 77 patients (44.0%) exhibited mixed CMBs involving two or more anatomical regions.

### Comparison of baseline characteristics between the CMB and non-CMB groups

Baseline characteristics differed between the non-CMB group (*n* = 318) and the CMB group (*n* = 175). Patients in the CMB group tended to be older than those in the non-CMB group (*p* = 0.006). In addition, vascular risk factors, including hypertension, previous ischemic stroke, and prior antiplatelet use, were more frequently observed in patients with CMBs (all *p* < 0.05). Several laboratory parameters also differed between groups. Total cholesterol and LDL-C levels were lower in patients with CMBs, whereas creatinine levels were higher (all *p* < 0.05). Of note, the proportion of high-grade IPN was significantly increased in the CMB group (*p* < 0.001), further supporting a possible link between carotid plaque activity and CMBs (Table 1).

**Table 1:** Baseline characteristics of patients between the CMB and non-CMB groups.

Variables	All patients (*n* = 493)	Non-CMB group (*n* = 318)	CMB group (*n* = 175)	*P* value
Demographics				
Age (years), mean ± SD	63.28 ± 5.83	62.75 ± 5.69	64.25 ± 5.97	0.006
Male sex, *n* (%)	274 (55.6)	169 (53.1)	105 (60.0)	0.143
Vascular risk factors, *n* (%)				
Hypertension	330 (66.9)	195 (61.3)	135 (77.1)	<0.001
Diabetes mellitus	140 (28.4)	83 (26.1)	57 (32.5)	0.327
Hyperlipidemia	145 (29.4)	89 (28.0)	56 (32.0)	0.350
Smoking	120 (24.3)	76 (23.9)	44 (25.1)	0.759
Alcohol consumption	68 (13.8)	41 (12.8)	27 (15.4)	0.435
History of ischemic stroke	127 (25.7)	64 (20.1)	63 (36.0)	<0.001
Antiplatelet use	169 (34.2)	94 (29.5)	75 (42.8)	0.003
Statin use	127 (25.8)	73 (22.9)	54 (30.8)	0.055
Baseline blood pressure (mmHg), mean ± SD				
Systolic blood pressure	140.59 ± 19.35	139.43 ± 19.23	142.69 ± 19.45	0.073
Diastolic blood pressure	81.72 ± 11.97	81.43 ± 11.67	82.25 ± 12.53	0.465
Laboratory findings, median (IQR)				
Total cholesterol (mmol/L)	4.15 (3.46–4.78)	4.23 (3.54–4.95)	4.00 (3.42–4.52)	0.001
Triglycerides (mmol/L)	1.41 (0.94–1.93)	1.46 (0.97–2.08)	1.31 (0.87–1.82)	0.071
LDL-C (mmol/L)	2.11 (1.56–2.71)	2.15 (1.60–2.84)	1.99 (1.49–2.53)	0.038
HDL-C (mmol/L)	1.21 (1.04–1.43)	1.23 (1.05–1.43)	1.19 (1.03–1.40)	0.277
Fasting glucose (mmol/L)	5.31 (4.80–6.36)	5.36 (4.89–6.38)	5.22 (4.70–6.14)	0.080
Fibrinogen (mmol/L)	3.18 (2.80–3.72)	3.17 (2.79–3.69)	3.24 (2.85–3.85)	0.104
Creatinine (mmol/L)	68.30 (58.25–81.50)	66.80 (57.98–78.48)	71.00 (61.50–88.20)	0.005
IPN grade, *n* (%)				<0.001
Low-grade IPN	230 (46.7)	174 (54.7)	56 (32.0)	
High-grade IPN	263 (53.3)	144 (45.3)	119 (68.0)	

SD, standard deviation; IQR, interquartile range; LDL-C, low-density lipoprotein cholesterol; HDL-C, high-density lipoprotein cholesterol; IPN, intraplaque neovascularization.

### Correlation between IPN and CMB burden

**Figure 4:**
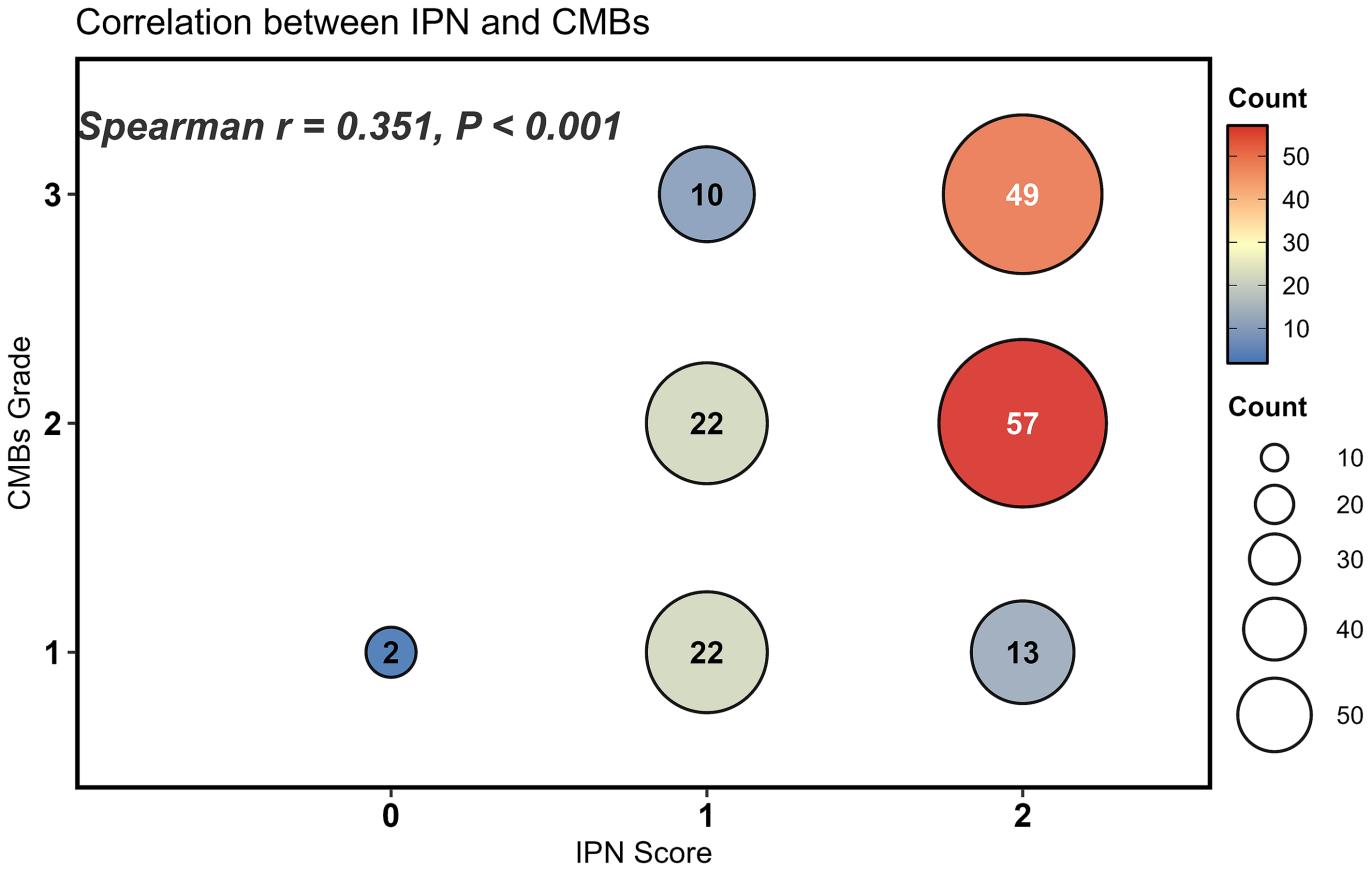
Correlation analysis of IPN score with severity of CMBs in patients.

Among the 175 patients with CMBs, IPN grades were distributed as follows: grade 0 (*n* = 2), grade 1 (*n* = 54), and grade 2 (*n* = 119), indicating a predominance of higher IPN grades in patients with CMBs. Spearman rank correlation analysis revealed a positive correlation between IPN grade and CMB severity (rs = 0.351, *p* < 0.001) (Figure 4), suggesting that higher IPN levels are associated with increased CMB severity. From a clinical perspective, increasing IPN grades may help identify patients at higher risk of more severe microvascular brain injury, thereby providing additional value for risk stratification beyond traditional vascular risk factors.

### Independent risk factors for CMBs

Variables with *p* < 0.05 in the univariate analysis were entered into a multivariable binary logistic regression model, with the non-CMB group used as the reference category. The results showed that age (OR = 1.056, 95% CI: 1.019–1.094, *p* = 0.003), high-grade IPN (OR = 2.793, 95% CI: 1.841–4.239, *p* < 0.001), hypertension (OR = 1.752, 95% CI: 1.098–2.795, *p* = 0.019), history of ischemic stroke (OR = 1.660, 95% CI: 1.047–2.631, *p* = 0.031), and antiplatelet use (OR = 2.198, 95% CI: 1.431–3.374, *p* < 0.001) were independent risk factors for the presence of CMBs (Table 2).

**Table 2:** Multivariable binary logistic regression analysis of risk factors for CMBs.

Variables	OR	95% CI for OR	*P* value
Age	1.056	1.019–1.094	0.003
High-grade IPN	2.793	1.841–4.239	<0.001
History of hypertension	1.752	1.098–2.795	0.019
Creatinine	1.003	0.996–1.010	0.404
Total cholesterol	0.840	0.597–1.180	0.315
LDL-C	1.041	0.684–1.584	0.851
History of ischemic stroke	1.660	1.047–2.631	0.031
Antiplatelet use	2.198	1.431–3.374	<0.001

### Independent risk factors for deep and infratentorial CMBs

Baseline characteristics were compared between the non-CMB group (*n* = 318) and the deep/infratentorial CMB group (*n* = 40). Patients with deep/infratentorial CMBs were older (*p* < 0.001) and had a higher proportion of high-grade IPN (*p* < 0.001), a higher prevalence of hypertension (*p* < 0.001), higher total cholesterol levels (*p* = 0.015), and more frequent antiplatelet use (*p* = 0.029). Multivariable binary logistic regression analysis demonstrated that age (*p* < 0.001), high-grade IPN (*p* < 0.001), hypertension (*p* < 0.001), and antiplatelet use (*p* = 0.016) were independent risk factors for deep/infratentorial CMBs (Table 3).

**Table 3:** Multivariable binary logistic regression analysis of risk factors for deep and infratentorial CMBs.

Variables	OR	95% CI for OR	*P* value
Age	1.136	1.072–1.202	<0.001
High-grade IPN	4.337	1.911–9.842	<0.001
History of hypertension	6.696	2.172–20.647	<0.001
Total cholesterol	0.844	0.573–1.243	0.390
Antiplatelet use	2.511	1.191–5.293	0.016

### Independent risk factors for lobar CMBs

Baseline characteristics were compared between the non-CMB group (*n* = 318) and the lobar CMB group (*n* = 58). Patients with lobar CMBs were older (*p* = 0.002) and had higher fibrinogen levels (*p* = 0.034), a higher prevalence of ischemic stroke (*p* < 0.001), and more frequent antiplatelet use (*p* < 0.001). To assess the contribution of carotid IPN and hypertension, these variables were included in a multivariable binary logistic regression model. The analysis demonstrated that age (*p* < 0.001), fibrinogen level (*p* = 0.003), history of ischemic stroke (*p* = 0.004), and antiplatelet use (*p* < 0.001) were independent risk factors for lobar CMBs. In contrast, high-grade IPN (OR = 1.638; 95% CI: 0.873–3.073; *p* = 0.124) and hypertension (OR = 1.577; 95% CI: 0.751–3.313; *p* = 0.229) were not significantly associated with an increased risk of lobar CMBs (Table 4).

**Table 4:** Multivariable binary logistic regression analysis of risk factors for lobar CMBs.

Variables	OR	95% CI for OR	*P* value
Age	1.097	1.044–1.152	<0.001
History of hypertension	1.577	0.751–3.313	0.229
High-grade IPN	1.638	0.873–3.073	0.124
Fibrinogen	1.676	1.185–2.369	0.003
History of ischemic stroke	2.638	1.356–5.131	0.004
Antiplatelet use	4.777	2.479–9.205	<0.001

## Discussion

In this study of 493 patients with carotid plaques, we found that IPN detected by Angio PLUS ultrasound was positively associated with the severity of CMBs. Compared with low-grade IPN, high-grade IPN was significantly associated with an increased risk of overall CMBs and deep/infratentorial CMBs, and was identified as an independent risk factor for both outcomes-whereas no significant association was observed with lobar CMBs.

Structural features of the carotid artery, such as intima media thickness, arterial stiffness, and plaque characteristics serve as accessible “windows” for investigating microvascular pathology that cannot be directly visualized. In a prospective community-based cohort, Ding et al. (18) demonstrated that ultrasound-derived markers of carotid atherosclerosis were associated with an 11, 14, and 13% increased risk of developing CMBs per standard deviation increase over a 5.2-year follow-up. After stratification by CMB location, these associations disappeared for lobar CMBs but were strengthened for deep and infratentorial CMBs (18, 24, and 23%, respectively). The authors proposed that carotid atherosclerosis may promote deep/infratentorial CMBs through hypertension-related small-vessel injury, whereas lobar CMBs may arise predominantly from cerebral amyloid angiopathy (CAA), suggesting distinct underlying pathogenic pathways.

A cross-sectional study of a Han Chinese population found that, after adjusting for confounding factors such as age, sex, cardiovascular risk, lacunar infarction, and white matter hyperintensities, carotid intima-media thickness (IMT) was also associated with deep and subcortical CMBs, but was not associated with lobar CMBs (19). This study extends these observations and found a correlation between the severity of carotid IPN and CMBs. Compared with patients with low IPN scores, those with high IPN scores had a 1.8-fold increased risk of CMBs and a 3.3-fold increased risk of deep and subcortical CMBs; however, no correlation was observed with the occurrence of lobar CMBs, providing supporting evidence for the aforementioned study findings. The selective association between IPN and deep/subtentorial CMBs may reflect distinct pathophysiological differences in the development of CMBs. Deep and subtentorial CMBs are primarily associated with hypertensive arterial lesions affecting small perforating arteries, which are highly susceptible to systemic atherosclerosis and hemodynamic stress (20). Therefore, as a high-level marker of plaque activity, IPN may indicate the presence of a pro-inflammatory microenvironment with microvascular damage (21), which primarily affects these pressure-sensitive vessels. In contrast, lobar CMBs is primarily driven by cerebral amyloid angiopathy, a pathogenic process that is largely independent of extracranial atherosclerosis and carotid plaque vulnerability (22).

To improve the ability to identify vulnerable plaques and guide clinical interventions, this study used plane-wave hypersensitive microflow imaging ultrasound to evaluate IPN in carotid artery plaques. IPN refers to new microvessels formed by the proliferation and migration of vascular endothelial cells on the basis of atherosclerotic lesions; these microvessels primarily originate from adventitial nourishing vessels. This process constitutes pathological angiogenesis, characterized by immature and structurally fragile microvessels extending from the adventitia toward the media and intima, gradually invading the base and shoulder regions of the plaque (23). A growing body of experimental evidence confirms that neovascularization from adventitial nourishing vessels and the resulting intraplaque hemorrhage represent critical stages in the formation of vulnerable atherosclerotic plaques (24, 25). Intraplague hemorrhage may be the ultimate pathological outcome of most IPN. In a study of patients who underwent carotid endarterectomy, intraplague hemorrhage in resected specimens was an independent risk factor for CMBs; the larger the extent of intraplague hemorrhage, the higher the risk of CMBs (26). It is worth noting that in this study, the prevalence of high-score IPN detected by Angio PLUS reached 68.0%, which is higher than the approximately 30% prevalence of vulnerable plaques reported in epidemiological surveys (27). This finding suggests that carotid atherosclerosis and plaque formation are associated with the development of intracranial CMBs even before intraplaque hemorrhage or the formation of vulnerable plaques.

The specific mechanisms linking carotid IPN and CMBs remain unclear. However, both conditions share several well-established vascular risk factors (such as aging, hypertension, and atherosclerosis). This commonality suggests that large-vessel and small-vessel diseases may be different manifestations of a shared process of vascular aging. Throughout the vascular aging process, pathological changes can occur throughout the entire arterial system—ranging from atherosclerosis and plaque formation in large arteries to small vessel occlusion and microaneurysm formation, ultimately progressing to CMBs. These diverse vascular phenotypes may reflect the body’s heterogeneous responses to common vascular damage. Therefore, carotid IPN can serve as a marker of widespread vascular damage affecting cerebral microcirculation. Elevated levels of reactive oxygen species (ROS) may play an important role in the progression of CMBs. One proposed pathway involves age-related depletion of nicotinamide adenine dinucleotide (NAD^+^), leading to impaired activity of the NAD^+^-dependent deacetylase SIRT1 and subsequent mitochondrial dysfunction, which is associated with increased mitochondrial ROS production (28). On one hand, excessive ROS promotes the initiation and progression of atherosclerosis through oxidative stress–mediated vascular injury (29). On the other hand, ROS may contribute to the development of CMBs by activating matrix metalloproteinases (MMPs), degrading extracellular matrix components, and weakening the structural integrity of the vascular wall (30). Furthermore, aging is characterized by a persistent state of chronic systemic low-grade inflammation, known as Inflammaging (31). Senescent cells produce a variety of pro-inflammatory mediators, collectively known as the senescence-associated secretory phenotype (SASP), which sustains chronic vascular inflammation (32). Among these, TNF-*α* released by senescent vascular smooth muscle cells promotes monocyte recruitment and contributes to carotid atherosclerosis (33). Meanwhile, inflammatory mediators such as IL-1β and IL-6 increase Blood–Brain Barrier (BBB) permeability by promoting tight junction protein degradation, leading to red blood cell extravasation and CMB formation (34). These aging-related mechanisms provide a plausible framework for elucidating the association between carotid IPN and CMBs and may help explain why age was consistently identified as an independent risk factor in our multivariable models. However, further mechanistic studies are required to clarify the underlying causal pathways.

This study has several important clinical implications. First, carotid IPN assessed by Angio PLUS, as a non-invasive and widely available ultrasound marker, may serve as a useful imaging biomarker for early identification of patients at higher risk of CMBs. This may help clinicians refine cerebrovascular risk stratification and guide more individualized management strategies, particularly in patients with multiple vascular risk factors. Second, our findings may have potential implications for clinical decision-making regarding antithrombotic therapy, as patients with high-grade IPN may require more cautious evaluation of bleeding risk.

The novelty of this study lies in the application of Angio PLUS–derived IPN as a surrogate marker of plaque neovascularization to investigate its relationship with CMB burden and location-specific CMB patterns. Unlike previous studies focusing mainly on traditional markers of carotid atherosclerosis such as intima-media thickness or stenosis severity.

This study has several limitations. First, as a cross-sectional study, causal relationships between carotid IPN and CMBs cannot be inferred. Second, since the study population consisted of inpatients in a single-center neurology department with a high prevalence of ischemic stroke and a history of antiplatelet therapy, there was a certain degree of selection bias. Finally, due to limitations in experimental conditions, inflammatory factors such as IL-1β and IL-6 could not be included in the regression model for adjustment, thereby reducing the model’s validity. In future studies, a stratified design could be adopted to include a larger sample size and establish a longitudinal cohort with regularly updated imaging data, with the aim of providing more robust evidence for the use of carotid IPN—an ultrasound imaging marker—in disease risk stratification and clinical treatment decision-making.

## Conclusion

This study demonstrates a positive association between carotid IPN, as detected by Angio PLUS, and the severity of CMBs. Compared with low-grade IPN, high-grade IPN was associated with an increased risk of overall CMBs and deep/infratentorial CMBs and emerged as an independent risk factor for both, whereas no significant association was observed with lobar CMBs. These findings suggest distinct pathophysiological mechanisms underlying CMBs in different anatomical locations.

## Data Availability

The raw data supporting the conclusions of this article will be made available by the authors, without undue reservation.
